# Reduced risk of synovial sarcoma in females: X-chromosome inactivation?

**DOI:** 10.1038/sj.bjc.6600362

**Published:** 2002-07-15

**Authors:** X Bu, L Bernstein, R K Brynes

**Affiliations:** Department of Pathology, University of Southern California, Keck School of Medicine, Los Angeles County+University of Southern California Medical Center, Los Angeles, California, USA; Department of Preventive Medicine, University of Southern California, Keck School of Medicine, Los Angeles, California, USA

**Keywords:** synovial sarcoma, X-chromosomal inactivation, reduced risk

## Abstract

Synovial sarcoma shows a characteristic t(X;18) translocation but not the expected female predominance in incidence. We speculate that, among females, one X-chromosome is inactivated and that only the translocation to an active X-chromosome leads to development of synovial sarcoma. Population-based cancer registry data from the SEER program support this hypothesis.

*British Journal of Cancer* (2002) **87**, 28–30. doi:10.1038/sj.bjc.6600362 www.bjcancer.com

© 2002 Cancer Research UK

## 

Synovial sarcoma (SS) is a malignant soft tissue tumour with a slight male predominance and accounts for approximately 7–10% of all soft tissue sarcomas. The histogenesis of this tumour is still unknown. The tumour can develop at any age (Weiss and Goldblum, 2001). According to United States National Cancer Institute Surveillance, Epidemiology, and End Results (SEER) data for 1973–1998 (2001), the incidence of this tumour is roughly one per million adults.

More than 95% of SS cases demonstrate a characteristic t(X;18) translocation involving the SYT gene on chromosome 18 and SSX genes on the X chromosome (dos Santos *et al*, 2001). Both SYT and SSX proteins appear to play a role in transcriptional regulation. The SSX gene family consists of at least six related and closely linked genes located at Xp11.2. Most cases of SS studied for the translocation have been found to have either SYT-SSX1 or SYT-SSX2 fusion transcripts. It has been shown that the translocation-derivative chromosome 18 or SSX-SYT fusion gene may be lost during tumour development (Smith *et al*, 1987; Molenaar *et al*, 1989), indicating that only the SYT-SSX fusion gene on the X-chromosome is essential for tumorigenesis.

Considering the high frequency and specificity of the t(X;18) translocation in SS, the SYT-SSX fusion gene is the likely causal mutation for SS. Since females have two X-chromosomes, we would expect that they would be twice as likely as males to develop a t(X;18) translocation. This observation has been made in alveolar soft part sarcoma, a female predominance tumour in which a specific t(X;17) translocation was recently identified (Ladanyi *et al*, 2001). However, the observed incidence rates of SS in the population-based SEER data do not reflect a female predominance. Instead the male-to-female ratio of age-standardised incidence rates is 1.1 : 1. This observation prompted us to investigate the possible role that X-inactivation plays in explaining this discordance.

Although the mechanism of X-inactivation remains largely a mystery, recent advances provide a better understanding of this phenomenon. The X inactive-specific transcript gene (Xist) located within the X-inactivation center (Xic) on the long arm of the X chromosome plays a central role in X-inactivation. This process is initiated in Xic and then spreads across the entire X-chromosome. X-inactivation can spread as far as 100 Mb into the attached autosomal segment of an X;autosome translocation (White *et al*, 1998). Inactivation can also spread into an autosome from Xist-containing transgenes (Lee *et al*, 1996). Since the available data show that the causal translocation fusion gene for SS is located on chromosome X (Smith *et al*, 1987; Molenaar *et al*, 1989), we hypothesise that those SYT-SSX fusion transcripts located on the inactivated X-chromosome are non-tumorigenic. This inactivation, therefore, reduces the likelihood of SS development in females to the same level as that of males and provides an explanation for the nearly balanced male : female ratio observed in SS. In the current study, we utilised SEER incidence data to test our hypothesis.

## MATERIALS AND METHODS

Age-specific and sex-specific SS incidence data (shown in Table 1

**Table 1:**
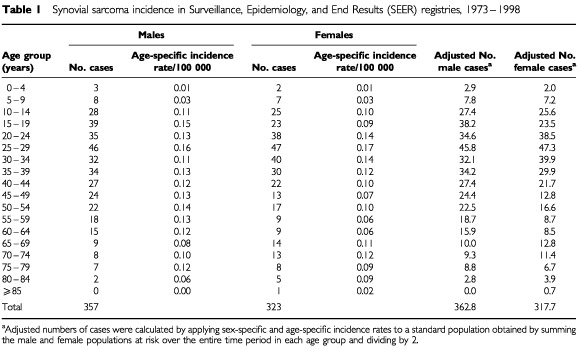
Synovial sarcoma incidence in Surveillance, Epidemiology, and End Results (SEER) registries, 1973–1998

) were derived from SEER Program Public-Use Data (2001). A total of 680 SS cases (ICDO morphology codes 9040, 9041, 9042, and 9043) were diagnosed in 11 SEER registry regions providing data for some or all of the years 1973 to 1998. Since the observed male and female population counts differ within each age group, we age-adjusted the observed male and female case counts in each age group (see footnote in Table 1). We summed the age-specific numbers to obtain a total adjusted count of cases for males and for females. Total observed male and female cases were then utilised to calculate the observed proportion. In the current study, we did not analyse the data according to race or geographical region due to the limited number of cases.

A Z test was used to determine whether the observed proportion of male cases (standardised to the average person-years distribution) differed significantly from the expected proportion based on the hypothesis of X-inactivation and the alternative competing hypothesis of no inactivation (see Appendix).

## RESULTS

Age-specific incidence rates of SS among males and females and the number of SS cases by sex in the 11 SEER registries providing data for some or all of the years 1973 to 1998 are presented in Table 1. The observed proportion of male cases standardised to the average population distribution for males and females is 362.8/(362.8+317.7)=53.3%. Under the hypothesis of X-inactivation, the expected proportion for male cases calculated from Equation 3 is 52% and the observed and expected proportions of male cases do not differ (Z=0.68 and *P*=0.5) (Appendix 1). In contrast, under the alternative competing hypothesis of no X-inactivation, the expected proportion for male cases is 35%, which differs significantly from the observed proportion (Z=10.0 and *P*<0.00001).

## DISCUSSION

Our hypothesis is conditional on one X-linked gene mutation functioning as the causal event for developing SS. This one X-linked gene mutation is biologically plausible and supported by available data, namely, the high frequency and specificity of the t(X;18) translocation in SS. Single chromosomal translocations resulting in malignancy have been observed in chronic myelogenous leukemia and acute promyelocytic leukemia. Studies of transgenic mice support the oncogenic nature of the products of both fusion transcripts (Melnick and Licht, 1999; Jaffe *et al*, 2001).

In the current study, we utilised population-based SEER data, which now represent about 14% of the US population, to test our hypothesis. Although several relatively large SS case series have been reported in the literature, they are not population based and, thus, are not appropriate for our analysis. Most of those case series come from several major tertiary care cancer hospitals and are subject to ascertainment bias.

X-inactivation can spread into the attached autosomal segment of an X;autosome translocation or into an autosome from Xist-containing transgenes (Lee *et al*, 1996; White *et al*, 1998). White *et al* (1998) showed that most of the translocated autosomal genes or expressed sequence tags (EST) are subject to inactivation, but a small percentage may escape. Although we have no evidence that the lesional SYT-SSX transcript falls into this latter category, the results from our study should be interpreted with caution. A well-designed molecular study may better delineate this issue. A recently reported X-inactivation profile of the human X-chromosome included the expression of 224 X-linked genes and ESTs by reverse-transcription-PCR analysis (Carrel *et al*, 1999). However, SSX genes were not included possibly because normal expression is limited to very few human tissues such as testis and thyroid or there are multiple copies of SSX genes (Crew *et al*, 1995; Fligman *et al*, 1995). However, all the flanking genes or ESTs of the SSX1 or SSX2 genes were subject to inactivation, further supporting our hypothesis.

In conclusion, we provide population-based evidence supporting a decisive role for X-inactivation in the fate of an oncogenic chromosomal translocation fusion transcript. Although females have approximately twice the risk for developing the t(X;18) translocation as do males, their actual risk for developing SS is similar to that of males probably due to X-inactivation. Confirmation of an X-inactivation hypothesis in SS must await studies at the molecular level.

## APPENDIX 1

### Calculation of the expected male and female proportions in SS cases.

In order to estimate the expected male and female proportions of SS cases, we first estimated the expected probabilities of developing t(X;18) translocations for males and females.

Should a chromosomal translocation develop in a cell, the conditional probabilities for such a translocation being a SYT-SSX translocation are:

and

where S, G, and C are target gene size, total human genome size, and constant, respectively, and m and f are males and females. A factor of two, for two copies of the S_SSX_ gene in females, is cancelled out by X-inactivation; therefore, this factor is not included. If we assume C_f_ =C_m_, then the expected proportion of male cases will be

where I=1 for the hypothesis of X-inactivation and I=2 for the alternative competing hypothesis of no X-inactivation. The total human genome sizes for males and females were obtained from the most recent publication of the Human Genome Project (International Human Genome Sequencing Consortium, 2001). Note that the terms on the right-hand side of the Equation 3 are only dependent on the male and female genome sizes. This statement theoretically holds for other chromosomal translocations.
